# Multicolor Whole-Cell Bacterial Sensing Using a Synchronous Fluorescence Spectroscopy-Based Approach

**DOI:** 10.1371/journal.pone.0122848

**Published:** 2015-03-30

**Authors:** Damien Parrello, Christian Mustin, David Brie, Sebastian Miron, Patrick Billard

**Affiliations:** 1 Université de Lorraine, CNRS, Laboratoire Interdisciplinaire des Environnements Continentaux (LIEC), UMR 7360, Vandoeuvre-lès-Nancy, France; 2 Université de Lorraine, CNRS, Centre de Recherche en Automatique de Nancy (CRAN) UMR 7039, Vandoeuvre-lès-Nancy, France; UC Berkeley, UNITED STATES

## Abstract

The wide collection of currently available fluorescent proteins (FPs) offers new possibilities for multicolor reporter gene-based studies of bacterial functions. However, the simultaneous use of multiple FPs is often limited by the bleed-through of their emission spectra. Here we introduce an original approach for detection and separation of multiple overlapping fluorescent signals from mixtures of bioreporters strains. The proposed method relies on the coupling of synchronous fluorescent spectroscopy (SFS) with blind spectral decomposition achieved by the Canonical Polyadic (CP) decomposition (also known as Candecomp/Parafac) of three-dimensional data arrays. Due to the substantial narrowing of FP emission spectra and sensitive detection of multiple FPs in a one-step scan, SFS reduced spectral overlap and improved the selectivity of the CP unmixing procedure. When tested on mixtures of labeled *E*. *coli* strains, the SFS/CP approach could easily extract the contribution of at least four overlapping FPs. Furthermore, it allowed to simultaneously monitor the expression of three iron responsive genes and pyoverdine production in *P*. *aeruginosa*. Implemented in a convenient microplate format, this multiplex fluorescent reporter method provides a useful tool to study complex processes with different variables in bacterial systems.

## Introduction

Fluorescent proteins (FPs) have become valuable tools for investigating biological processes in living cells. Their unique ability to emit fluorescence in both eukaryotic and prokaryotic cells with no substrate or cofactor requirement makes them suitable biochemical markers for a variety of biological applications. FPs are regularly used, for instance, as reporters for non-invasive monitoring of gene expression as well as for studying subcellular localization and dynamics of proteins [1]. In ecological studies, they can serve as tags to track bacteria in complex environmental matrices such as biofilms or soils, and to survey mutualistic or pathogenic associations that bacteria form with plants or animals [2]. Another broad application area is the development of whole cell bacterial sensors, which typically relies on gene fusions between a stress responsive promoter and a reporter gene (e.g., green fluorescence protein (GFP)). With the advances in the emerging field of synthetic biology, FPs are now commonly used as output reporters to quantify the dynamic behavior of biological components from artificial genetic circuits [3][4].

The last decade has seen the emergence of a wide variety of new FP variants with fluorescence emission ranging from blue to near infrared and featuring, even in a same spectral category, improved photophysical properties including brightness, fluorophore maturation rate and photostability. The availability of such an ever growing collection of FPs offers the opportunity for multicolor labeling studies, where different components of biological systems and their interactions can be monitored simultaneously by mean of micro- or spectroscopic technics [5][6]. However, the combined use of multiple FPs is often limited by the overlaps among their fluorescence spectra. While the simultaneous detection of two or three spectrally distant FPs is commonly achieved using appropriate optical filters, significant spectral overlaps inevitably appear as the number of FPs increases, thereby complicating the accurate separation of individual signals in the mixture. In fluorescence imaging, this limitation can be overcome through linear unmixing approaches, which determine the contribution of each fluorophore in the overall fluorescence data, assuming that the mixed spectrum is a the linear combination of the reference spectra of all involved fluorophores [7][8]. To do so, the spectral properties of each spectrum component must be known for reliable spectral unmixing. This is however rarely the case in biological systems since the spectra of fluorophores are subject to experimental and biological fluctuations (e.g. cell type, pH, temperature) and may differ from the reference ones. Furthermore, inherent cellular autofluorescence and background fluorescence should be defined spectrally before unmixing calculation to be substracted from the actual fluorophore signals. Several alternative methods allowing blind spectral separation of multiple unknown fluorescent sources have been proposed, among which we may cite Principal Component Analysis (PCA) or Non Negative Matrix Factorization (NMF) [9]. A multidimensional extension of these bilinear decompositions is the Canonical Polyadic (CP) decomposition or Candecomp/Parafac [10][11] which has the advantage of yielding a unique solution in most cases [12][13][14]. This uniqueness feature, along with the blindness of the algorithm makes CP an attractive exploratory tool that generally provides significant insights about the underlying structure of the spectral data. While the method has gained interest in chemometrics and food technology, applications to quantify fluorophores in biological systems are still scarce [15].

In this context, the present study aimed at developing a method for the recording and analysis of multiple fluorescent signals from mixtures of whole cell bioreporters. The approach relies on the acquisition of spectra by synchronous fluorescence spectroscopy (SFS) combined with blind CP decomposition of the fluorescence data to discriminate FP signals from autofluorescence or extrinsic fluorescent compounds. Recording fluorescence spectra through synchronous scanning of both excitation and emission wavelengths is known to provide narrower and more symmetric spectra in a wider spectral range [16]. This scan functionality results in a higher spectral selectivity for the monitoring of fluorescence sources. By reducing spectral bleed through, SFS serves as a simple and useful method for the simultaneous determination of fluorescent components in complex mixtures [17]. In the present work, we first applied the combined SFS/CP approach to mixtures of *E*. *coli* strains expressing fluorescent proteins with overlapping spectra. The method was then used to monitor the expression of iron responsive genes and siderophore production in *Pseudomonas aeruginosa*.

## Materials and Methods

### Bacterial strains, plasmids and growth conditions

Bacterial strains and plasmids used in this study are listed in Table 1. Bacteria were routinely grown aerobically on LB agar or in LB broth. Deferrated casamino acids medium (DCAA medium [18]) was used in iron sensing assays with *Pseudomonas aeruginosa*. *E*. *coli* and *P*. *aeruginosa* were grown at 37°C. When required, the medium was supplemented with kanamycin at final concentrations of 40 μg/ml (for *E*. *coli*) or 250 μg/ml (for *P*. *aeruginosa*).

**Table 1:** Bacterial strains and plasmids.

**Strains and plasmids**	**Relevant characteristic**	**Source or reference**
Bacterial strains		
*E*. *coli* TOP10	F^-^ *endA1 recA1 galE15 galK16 nupG rpsL* (Str^R^) Δ*lacX74* Φ80*lacZ*Δ*M15 araD139* Δ(*ara*,*leu*)*7697 mcrA* Δ(*mrr-hsdRMS-mcrBC*) λ^-^	Invitrogen
*P*. *aeruginosa* PAO1	Wild type	ATCC 15692
*P*. *aeruginosa* PAO1 Δ*pvdA*	Pyoverdine-deficient mutant	[19]
Plasmids^a^		
pPROBE’-GFP[LVA]	Broad host-range promoter probe vector; Km^r^	[20]
pPROBE-NT’	Broad host-range *gfp* promoter probe vector ; Km^r^	[20]
pJBA28	Source of *P*_*A1/O4/O3*_ promoter; Ap^r^; Km^r^	[21]
pPROBE-NT’lac	pPROBE-NT’ derivative with *gfp* driven by *P*_*A1/O4/O3;*_ Km^r^	This study
pmcherry	Source of mCherry coding sequence; Ap^r^	Clontech
pE2-Orange-N1	Source of E2-Orange coding sequence; Km^r^	[22]
pPBY538	Promoter probe plasmid carrying *E*. *coli* codon optimized *TurboYFP*; Km^r^	This study
pPBO561	Promoter probe plasmid carrying *e2-orange*; Km^r^	This study
pPBR591	Promoter probe plasmid carrying *E*. *coli* codon optimized *dsred-express2*; Km^r^	This study
pPBR610	Promoter probe plasmid carrying *mcherry*; Km^r^	This study
pPB-lac-Y538	pPBY538 carrying a *P*_*A1/O4/O3-*_ *turbo-yfp* fusion; Km^r^	This study
pPB-lac-O561	pPBO561 carrying a *P*_*A1/O4/O3-*_ *e2-orange* fusion; Km^r^	This study
pPB-lac-R591	pPBR591 carrying a *P*_*A1/O4/O3-*_ *dsred-express2* fusion; Km^r^	This study
pPB-lac-R610	pPBR610 carrying a *P*_*A1/O4/O3-*_ *mcherry* fusion; Km^r^	This study
pPB-bfrB-O561	pPBO561 carrying a *bfrB*-*e2-orange* fusion; Km^r^	This study
pPB-pvdA-R591	pPBR591 carrying a *pvdA*-*dsred-express2* fusion; Km^r^	This study

^a^ Individual plasmid names in the set of pPB vectors have the form pPBXyyy, where X and yyy denote the spectral class and the fluorescence emission maximum (in nm) of the encoded FP, respectively.

Detailed methods for construction of plasmids used in this work are described in the supporting S1 Text. Briefly, the fluorescent reporter pPB plasmid series was obtained by replacing the *gfp* gene of pPROBE’-*gfp*[LVA] [20] by genes coding 4 different fluorescent reporters (TurboYFP, E2-Orange, DsRedExpress and mCherry). A second set of vectors designated pPB-lac was also constructed by inserting the LacI-repressible *P*_*A1/O4/O3*_ promoter [23] upstream each reporter gene in pPB plasmids.

### General DNA manipulation

DNA manipulations were performed following standard molecular biology techniques. Enzymes were purchased from Thermo Scientific. Plasmids extraction and DNA cleanup were performed by using Nucleospin miniprep and Nucleospin gel and PCR cleanup columns (Macherey Nagel), respectively. PCR primers were purchased from Eurofins Genomics (Ebersberg, Germany). PCR amplifications were carried out with DreamTaq DNA Polymerase from Thermo Scientific. All constructs were verified by DNA sequencing (Eurofins Genomics). Plasmids were introduced in *P*. *aeruginosa* by electroporation following the method of Choi et al. [24].

### Mixture of different fluorescent *E*. *coli* strains

Serial dilutions of cell suspensions were obtained from four different *E*. *coli* TOP10 strains, each expressing a particular fluorescent protein (GFP, TurboYFP, E2-Orange and DsRed-Express2) constitutively. Cell suspensions were mixed in defined ratios in black 96-well microplates (Eppendorf Microplate 96/V), with a mixing pattern designed to generate a trilinear data set of four overlapping fluorescent signals useable by the signal processing method (Candecomp/Parafac) described below. Mixtures were prepared using an automated pipetting system (Eppendorf epMotion 5070) as follows: overnight LB cultures were washed twice in NaCl 0.8% and cell density was adjusted to OD_600_ = 2 (approx. 8.10^8^ cells/ml). First, cell suspensions of either TOP10/pPROBE-NT’Lac (GFP) or TOP10/pPB-lac-Y538 (TurboYFP) were 1.65-fold serially diluted six times in a deepwell plate (Eppendorf) until reaching a 20 times dilution (1.65^6^). Similarly, TOP10/pPB-lac-O561 (E2-Orange) and TOP10/pPB-lac-R591 (DsRed-Express2) were diluted in the same plate but using a 3-fold serial dilution to avoid collinearity between the two sets of dilution. The untransformed TOP10 strain was used as a diluent to maintain a constant cell concentration in the wells. Diluted cell suspensions were then mixed in a second microplate following the ratios indicated S1 Fig. From column 1 to 7 the concentration of fluorescents reporters varied whereas from line A to E the ratios between reporters varied, while the cell density remained constant (OD_600_ = 2). Thus, the contribution of each fluorescent protein decreased or increased as a function of the dilution rank or of the mixing ratio. As a benchmark, microplates were prepared following the same pattern for each fluorescent strain mixed with untransformed TOP10 strains.

### Mixture of *P*. *aeruginosa* iron bioreporter strains

Overnight LB cultures of the two iron bioreporters *P*. *aeruginosa* PAO1/pPB-bfrB-O561 and PAO1/pPB-pvdA-R591 were washed twice in DCAA medium and suspended in the same medium to an OD_600_ of 0.1. The two cell suspensions were then mixed in a black polypropylene 96-well microplate and supplemented with different dilution of FeCl_3_ with an automated pipetting system (Eppendorf epMotion 5070) in a final volume of 200 μl as indicated in S2 Fig. Briefly, from column 1 to 12, iron concentration serially decreased 3-fold, from 2 mM FeCl_3_ (column 1) to 11 nM FeCl_3_ (column 12). From lane A to G, we linearly varied the ratio of bioreporter suspensions from 95% of PAO1/pPB-bfrB-O561 and 5% of PAO1/pPB-pvdA-R591 (line A) to 5% of PAO1/pPB-bfrB-O561 and 95% of PAO1/pPB-pvdA-R591 (lane G). The microplate was incubated at 37°C with shaking (130 rpm) under humid atmosphere to limit evaporation for 48h before fluorescence measurement.

A second experiment was performed with an additional iron bioreporter strain, PAO1/pPROBE-NT’-pvdS, following a similar procedure, except that cell suspensions were mixed in a final volume of 1 ml in 48-well microplates and incubated with 9 different iron concentrations as detailed in S3 Fig.

### Synchronous Fluorescence Spectra acquisition

Before fluorescence reading, cell suspensions were briefly homogenized with a MixMate vortex mixer (Eppendorf). Synchronous fluorescence spectra (SFS) were performed in 96 well microplates using a two-grating monochromator spectrofluorometer FLX-Xenius (SAFAS, Monaco), equipped with a 150 W Xenon lamp as the excitation source. The SFS analysis of cell suspensions was measured in the excitation wavelength range of 400–700 nm at a constant offset value ∆λ = λ_em_- λ_ex_ = 20 nm, which gave the best sensitivity and sharpest peaks among the tested offsets (i.e. 15, 20, 30, 40 nm). Spectra were recorded with a spectral step of 2 nm and a mean scan speed of 600 nm.min^-1^. The excitation and emission slits width were 10 nm and the photomultiplier voltage set at values between 650 V. Raw fluorescence signals (without filtering or smoothing) collected form mixtures of *E*. *coli* and *P*. *aeruginosa* and exported for further signal processing with Candecomp/Parafac algorithms running under Matlab software.

Synchronous spectrum of purified pyoverdine (Sigma-Aldrich, catalog no. P8124) was measured in a similar way in DCAA medium.

Overnight cultures of *E*. *coli* TOP10 expressing FP constitutively were also analyzed by synchronous fluorescence spectroscopy for a comparative analysis of *in vivo* FP brightness. Following spectra acquisition, the *in vivo* brightness was estimated from the fluorescence maximum intensity normalized to cell density.

### Candecomp/Parafac (CP) decomposition

The multi-way analysis of spectral data by CP algorithm provided multilinear decomposition of a data matrix without any a priori information about the FP spectra. In this work, it was assumed that synchronous fluorescent spectra coming from different batches are indicative of various mixtures of R fluorescent components. Each of the R fluorescent component spectrum, termed as the *r* ^th^ source (*r* = 1,…,R), is mathematically represented by a vector **s**_*r*_ = [*s*_*r*1_ … *s_rN_*]^*T*^ made of *N* entries, i.e. the fluorescence intensities at each wavelength. Thus, the acquisition of SFS spectra as a function of two crossed parameters (*M* x *P* values; e.g. biosensors ratio x iron concentration) generated a three-way data array X (three-order tensor; e.g. biosensors ratio x iron concentration x wavelength), which can be expressed by the following tri-linear CP mode:

$$X_{m,p,n}=\overset{R}{\underset{r=1}{\sum }}a_{rm}\;\cdot \;b_{rp}\;\cdot \;s_{rn}+E_{m,p,n}$$

where R is the number of fluorescence sources (i.e. decomposition rank). E is the residual error term (three-order tensor) including experimental error, signal noise or ‘non-linear’ component behavior. An equivalent representation is given by the following equation:

X=[[A,B,S]]+E

where the three matrices **A** (*M*-by-*R*), **B** (*P*-by-*R*) and **S** (*N*-by-*R*) are respectively obtained by stacking the vectors **a**_*r*_, **b**_*r*_ and **s**_*r*_. The main interest of the CP decomposition comes from its good uniqueness properties [12], meaning that the matrices **A, B** and **S** can be uniquely estimated from the data X under mild conditions. Moreover, even in difficult situations such as the presence of collinear vector in one mode (matrix), the addition of supplementary mathematical constraints such as the positivity of the different matrix entries, coupled with unimode uniqueness results of Guo et al. [14] yields a unique non negative CP decomposition. After selecting the expected number of fluorescent sources (R), the estimation of the unknown three matrices (**A**, **B** and **S**) was achieved by a non-negative alternating least-squares algorithm with a random initialization of the three matrices. The code implemented herein was developed by Bro [11] and is available in the Matlab N-way toolbox.

With SFS data set presented herein, performing the tri-linear CP decomposition yields the three matrices **S** (*N*-by-*R* matrix), **A** (*M*-by-*R* matrix), **B** (*P*-by-*R* matrix) representing respectively estimates of the *R* source spectra and the corresponding parameters profiles to the *M* and *P* values of the two parameters. The rank *R* of the decomposition (i.e. the number of sources *R*) is assessed iteratively by observing the decrease of the energy of the fitting error E as the number of components is increasing.

## Results and Discussion

### Characterization of FP labeled *E*. *coli* cells by synchronous fluorescence spectroscopy

**Fig 1:**
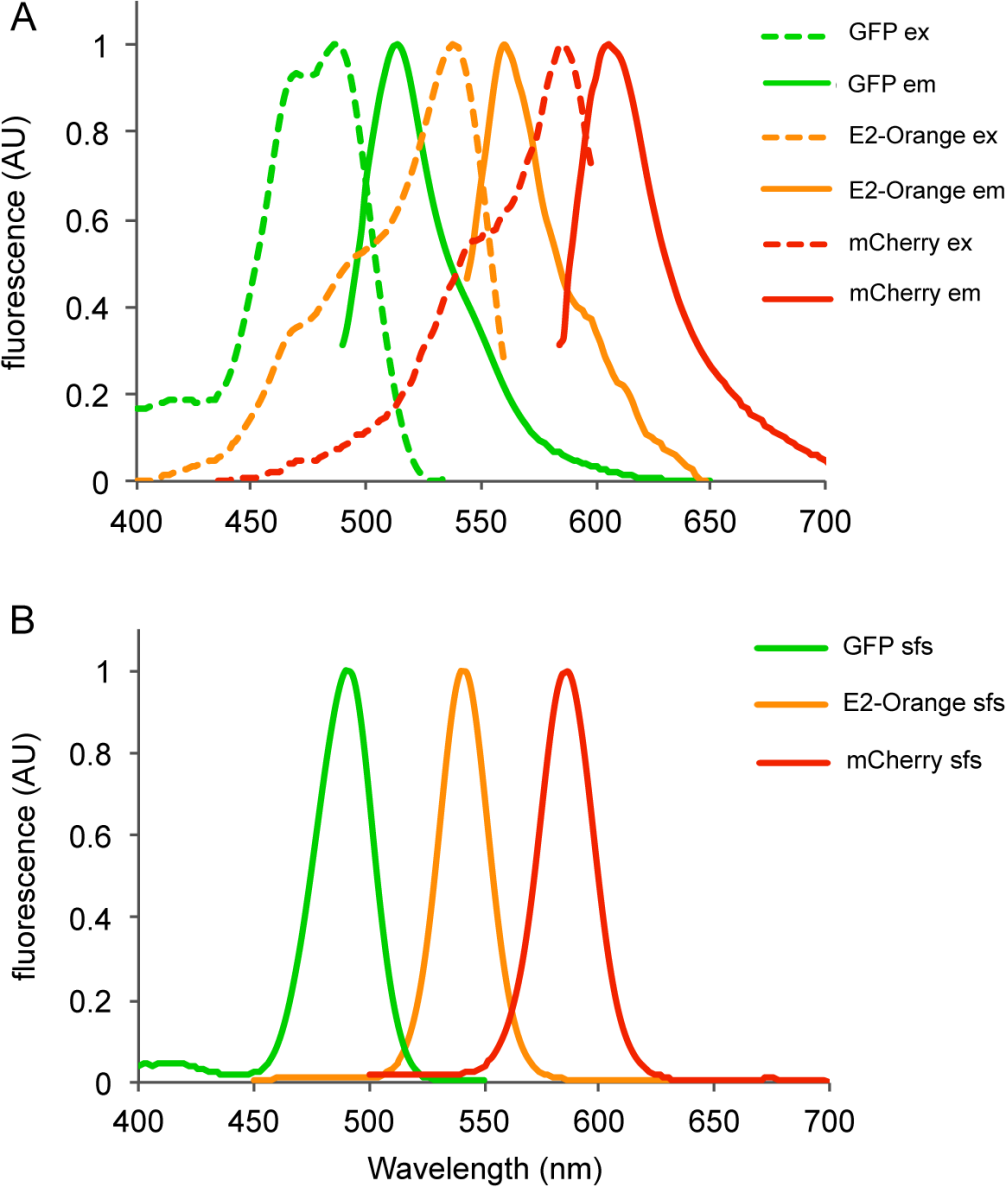
Fluorescence spectra of GFP, E2-Orange and mCherry. Shown are normalized excitation and emission (A) and synchronous (B) fluorescence spectra of *E*. *coli* cultures expressing GFP, E2-Orange and mCherry.

Synchronous fluorescence spectroscopy (SFS) is an increasingly popular tool for analyzing complex mixtures of fluorescent compounds. The combination of SFS with appropriate mathematical data processing tools enables the decomposition of complex fluorescent spectra into their components. To assess the gain provided by SFS compared to conventional fluorescent spectroscopy in differentiating mixtures of fluorescently labeled bacteria, we first recorded the fluorescent spectra of *E*. *coli* TOP10/pPB-lac by synchronous scanning and compared them to excitation and emission spectra. The pPB-lac plasmids are derivatives of the broad host range promoter-probe vector pPROBE’-GFP[LVA][20] utilizing different fluorescent reporter genes controlled by the strong *lac* promoter P_*A1/O4/O3*_, which is constitutive in *E*. *coli* TOP10. In the example provided (Fig 1), GFP, E2-Orange and mCherry exhibit relatively broad and asymmetrical excitation-emission spectral profiles with a significant degree of overlap. In contrast, the fluorescence signal in the synchronous spectra is confined in Gaussian shaped and much narrow peaks, which result in lower spectral overlap between all three FPs. This spectral simplification and bandwidth narrowing also holds for TurboYFP and DsRed-Express2 (as well as for other recent FPs we examined, e.g. mTagBFP2, Clover, E2-Crimson or mCardinal2; data not shown) as indicated by the systematic reduction of full width at half maximum (FWHM) of spectra acquired synchronously compared to emission peaks (Table 2). It is also noteworthy that the peak position, bandwidth and intensity in synchronous spectra depend on the wavelength offset ∆λ (i.e. fixed wavelength interval between excitation and emission monochromators). Therefore, the ∆λ can be tuned in order to amplify signals of interest or to limit interferences. Generally, we observed that fluorescence maxima shifted to shorter wavelengths with increasing ∆λ, and sharper and narrow peaks were obtained for ∆λ ranging from 15 to 30 nm. For the rest of this work, we chose a ∆λ = 20 nm which corresponds roughly to the mean Stokes shift (i.e. the difference between absorption and emission maxima) of the studied fluorescent proteins (Table 2).

**Table 2:** Properties of fluorescent proteins *in vitro* and in living *E*. *coli* cells.

Protein	λ_ex_ ^a^	λ_em_ ^a^	QY^b^	Bright-ness ^b^	Matu-ration^c^	λ_SFS_ ^d^	FWHM SFS ^e^	FWHM em. ^f^	SFS peak height ^g^	Reference ^h^
GFP	488	507	0.60	34	0.3	490	28	31	80	[25]
TurboYFP	525	538	0.53	56	ND	519	22	33	130	Evrogen
E2-Orange	540	561	0.54	20	1.3	540	24	39	40	[22]
DsRed-Express2	554	591	0.42	15	0.7	558	27	48	35	[26]
mCherry	587	610	0.22	16	0.7	584	29	44	11	[27]

^a^ λ_ex_ and λ_em_ are the excitation and emission maxima in nm, respectively

^b^ QY is the quantum yield; brightness is the product of QY and extinction coefficient (not provided).

^c^ Time in hour for fluorescence to reach half-maximal value after exposure to oxygen

^d^ Maxima of synchronous fluorescent spectra at a constant offset value ∆λ = 20 nm

^e^ Full-Width Half-Maximum of synchronous fluorescent spectra acquired at ∆λ = 20 nm

^f^ FWHM of emission spectra at absorption maxima

^g^ Fluorescence peak intensity for synchronous scan performed at ∆λ = 20 nm, expressed as relative fluorescence units normalized to cell density. The photomultiplier detector operated at a voltage of 650V.

Fluorescence data for ^d, e, f^ and ^g^ were obtained from cultures of *E*. *coli* TOP10 expressing FP constitutively.

^h^ Source of data for ^a, b^ and ^c^.

Synchronous scans of *E*. *coli* cultures expressing different FPs were performed at this offset and with a fixed signal gain to compare their brightness *in vivo*. The best performances were observed for TurboYFP, which was approximately 1.6-fold brighter than GFP, while mCherry was the dimmest FP with only one eighth the brightness of GFP. It is worth mentioning here that the measured *in vivo* fluorescence levels were strongly correlated (R^2^ = 0.95) with the *in vitro* FP brightness (Table 2), suggesting that, for the conditions tested, other factors such as mRNA stability or translation efficiency do not significantly affect FP expression. This observation also indicates that SFS may provide a simple way of estimating the relative *in vivo* brightness of FPs from different spectral classes, which is difficult to achieve through traditional spectroscopic or microscopic measurement [5].

### Coupling synchronous fluorescence spectroscopy to Candecomp/ Parafac decomposition method

**Fig 2:**
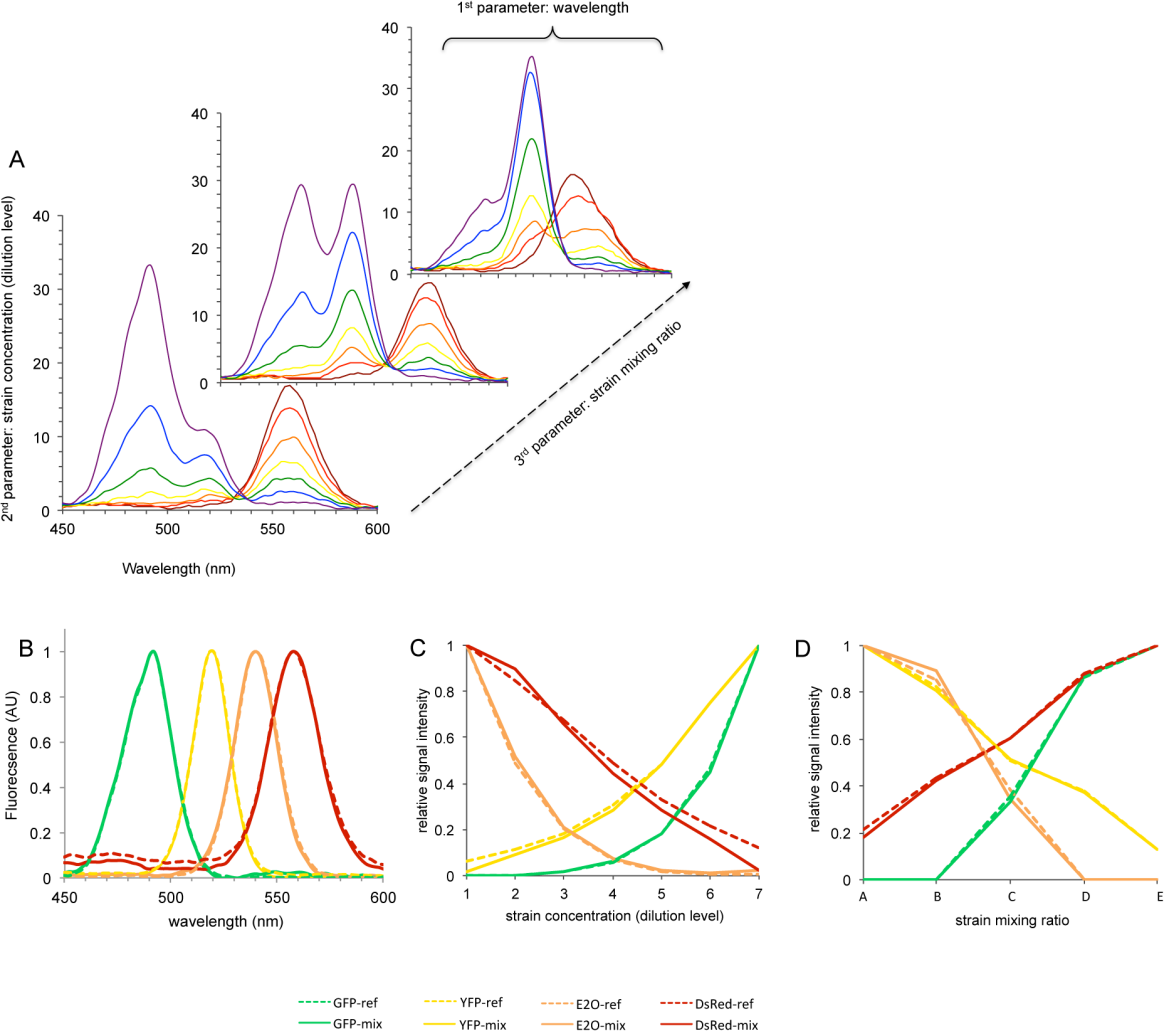
Synchronous spectra acquisition and CP decomposition of fluorescence from mixtures of four *E*. *coli* TOP10 strains expressing different FPs constitutively. (A) Typical SFS dataset of FP labeled strains mixtures (excerpt of 11 spectra out of 35). (B-D) Outcomes of CP decomposition. The fluorescence profiles estimated from CP analysis of mixtures of FP labeled strains (solid lines) are compared to those obtained from single labeled strains benchmarks (dashed lines). (B) Fluorescence profile as a function of labeled strain concentration (dilution level). (C) Synchronous fluorescent spectra. (D) Fluorescence profile as a function of strains mixing ratio. The mixture pattern for this experiment is presented in S1 Fig. YFP: TurboYFP; E2O: E2-Orange; DsRed: DsRed-express2.

The substantial bandwidth narrowing obtained by SFS suggested it could facilitate the extraction of individual signals in complex fluorescent mixtures. We therefore constructed such complex fluorescence signals by mixing four different TOP10 *E*. *coli* strains expressing FPs (GFP, TurboYFP, E2-Orange or DsRed-Express2) constitutively, and simulated variation of FP expression using different strain ratios and strain concentrations (see material and methods for detailed dilution protocol). Next, we applied the SFS procedure to each mixture and tested the ability of Candecomp/Parafac (CP) algorithm to extract the contribution of individual FPs. Altogether, the trilinear dataset of four overlapping fluorescence signals could be decomposed and compared to benchmark experiments involving single labeled strains treated in the same conditions, as depicted in Fig 2. Before CP decomposition, the fluorescence dataset appeared as a pool of 35 SFS multi-peak records (Fig 2A). The CP blind decomposition clearly shows that (i) the four fluorescence sources in the mixture could be reliably identified, as judged by the estimated spectra that nearly superimposed with those of the four FPs tested individually (Fig 2B), and (ii) the estimated sources of fluorescence in the mixture behave almost exactly as did fluorescence from individual FP reporter strains with the same level of dilution and relative concentration (Fig 2C and 2D, respectively). These results show that the coupled SFS/CP approach could solve the issue of FP bleed-through in multicolor labeling experiments, even in scenarios involving high spectral overlapping FPs such as E2-Orange and DsRed-Express2, whose synchronous fluorescence peaks are separated by only 18 nm (Table 2).

### Application of the joint SFS/CP approach to study iron homeostasis in *P*. *aeruginosa*

**Fig 3:**
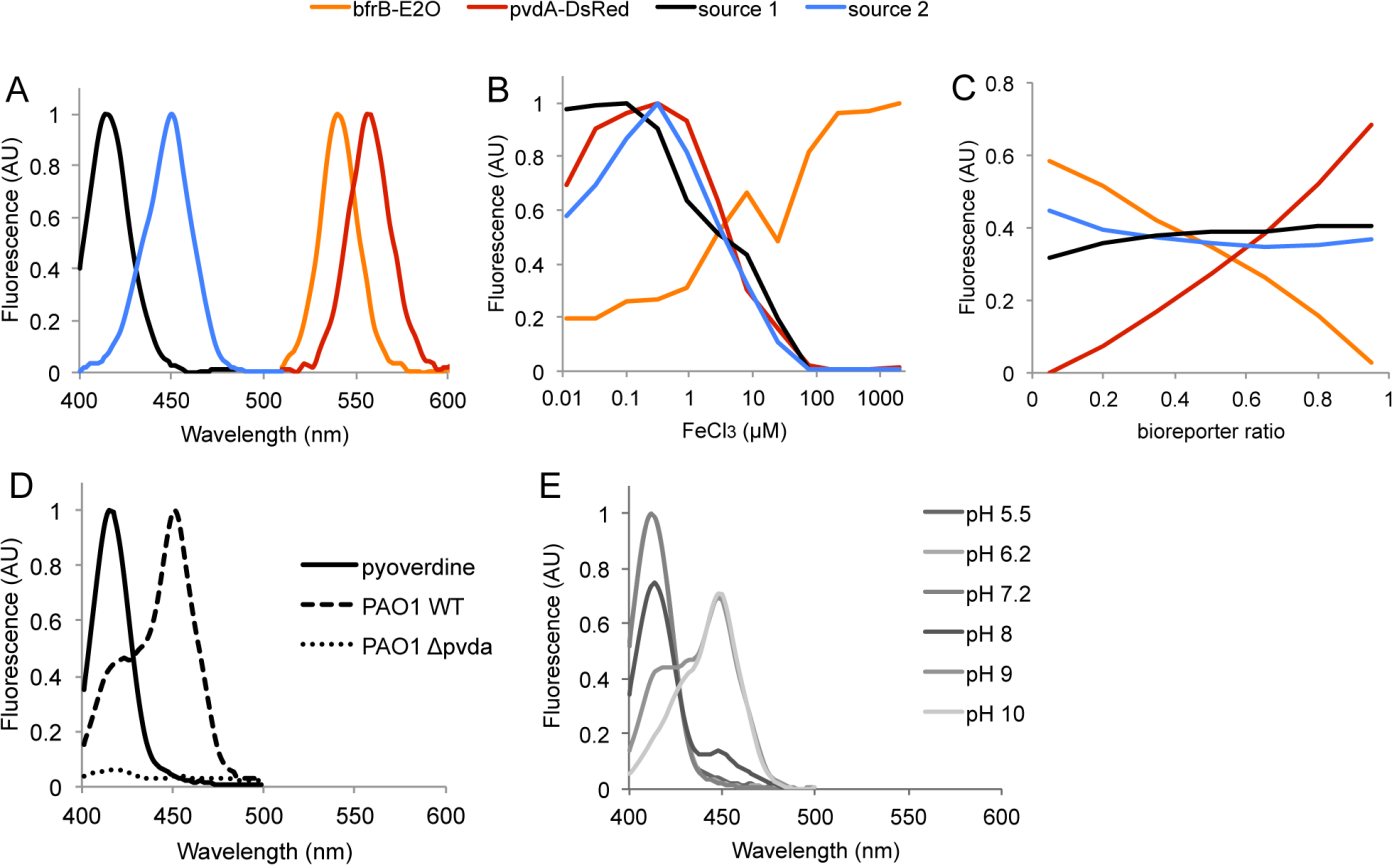
Spectral decomposition of fluorescence from mixtures of two *P*. *aeruginosa* PAO1 iron bioreporter strains harboring *pvdA*-*dsred-express2* and *bfrB-e2-orange* fusions. Shown are (A) the spectra of the four fluorescent sources identified from CP analysis and their profile as a function of (B) iron concentration and (C) the ratio of bioreporter strains in the mixture. The mixture pattern for this experiment is presented in S2 Fig. (D) Synchronous spectra of purified pyoverdine (solid line) and cultures of PAO1 wild type (WT, dashed line) and PAO1 ∆*pvdA* (large dashed lines) cells grown in low-iron DCAA medium. The later spectrum was normalized with respect to that obtained for PAO1 wild type. (E) Synchronous spectra of WT PAO1 culture supernatant as a function of pH. The DCAA growth medium supernatant was diluted tenfold in different buffers with pH adjusted to 5.2 and 6.2 (40 mM MES), 7.4 (40 mM MOPS) and 8, 9 and 10 (40 mM Tris-HCl).

Having demonstrated the proof of principle of SFS/CP on artificial mixtures of labeled bacteria, we tested its usefulness through the monitoring of combined *P*. *aeruginosa* iron bioreporters. Like most bacteria, *P*. *aeruginosa* must tightly regulate cellular iron acquisition and storage to prevent the deleterious effects of iron deficiency and iron excess [28]. Under iron restriction condition, *P*. *aeruginosa* produces two high-affinity iron chelating siderophores named pyoverdine and pyochelin that serve to deliver iron to the cell. The important background fluorescence noise caused by these siderophores prompted us to assess the relevance of our SFS/CP methodology. We then investigated the behavior of the *pvdA* and *bfrB* genes, two iron responsive genes from *P*. *aeruginosa* that display opposite iron-dependent transcriptional regulation. The *pvdA* gene encoding a L-Ornithine N^5^-oxygenase involved in an early step of the pyoverdine synthesis is repressed by iron. In contrast, the expression of the bacterioferritin encoding gene *bfrB* is induced under iron replete condition. Two *bfrB*-*e2-orange* and *pvdA*-*dsred-express2* gene fusions were constructed in pPB plasmids and introduced into *P*. *aeruginosa* PAO1. The resulting iron bioreporter strains were mixed in defined ratios and grown in the presence of different iron concentrations before SFS acquisition. From CP decomposition of the spectral data, four fluorescent sources could be identified: two of them unambiguously corresponded to E2-Orange and DsRed-Express2, the two others peaked at 416 nm and 450 nm (Fig 3A). The contribution of E2-Orange to the overall fluorescence increased with iron concentration, whereas the DsRed-Express2 signal increased upon iron depletion (Fig 3B), which is well in line with previously described expression of *pvdA* and *bfrB* genes from PAO1 [29],[30]. However, the iron-dependent induction profile of *bfrB-e2-orange* showed a modest yet reproducible decrease around 25 μM FeCl_3_. This particular behavior may reflect subtle regulation of bacterioferritin gene expression that would deserve further investigations. The two other fluorescence sources behave similarly as the expression profile of *pvdA-dsred-express2* with respect to iron concentration (Fig 3B) and were almost unaffected by variation of the bioreporter strains ratios (Fig 3C), suggesting they could correspond to one or two of the siderophores pyoverdine or pyochelin. A SFS control experiment carried out on strain *P*. *aeruginosa* PAO1∆*pvdA* lacking pyoverdine revealed the complete disappearance of the two signals, thus ruling out the possible contribution of pyochelin (Fig 3A and 3D). Interestingly, the supernatant of PAO1 WT cultures showed a SFS peak that shifted from 416 nm at acidic pH to 450 nm at pH 10 (Fig 3E). In contrast, synchronous spectra of purified pyoverdine exhibited a single band at 416 nm peak whatever the pH. These data strongly suggest that both sources actually correspond to two fluorescent forms of pyoverdine (PVD) we designated af-PVD (“acidic form”, 416-nm peak) and bf-PVD (“basic form”, 450 nm peak). While af-PVD most probably corresponds to iron-free pyoverdine, the nature of bf-PVD is still uncertain. Romanowski et al. [31] also observed a major fluorescent peak at around 490 nm in addition to the characteristic emission band of pyoverdine in phosphate-limited cultures of PAO1, however its origin was not investigated. A possible explanation for this fluorescent signal would be the particular pH-dependent biotic or abiotic interactions that pyoverdine can have with components of the local microenvironment [32].

The SFS/CP approach used here enabled to monitor reliably and simultaneously the expression of the *bfrB* and *pvdA* genes along with pyoverdine production, thus proving again its ability to integrate multiple signals from combinations of fluorescent whole cell bioreporters.

### Testing the limits of the SFS/CP approach

**Fig 4:**
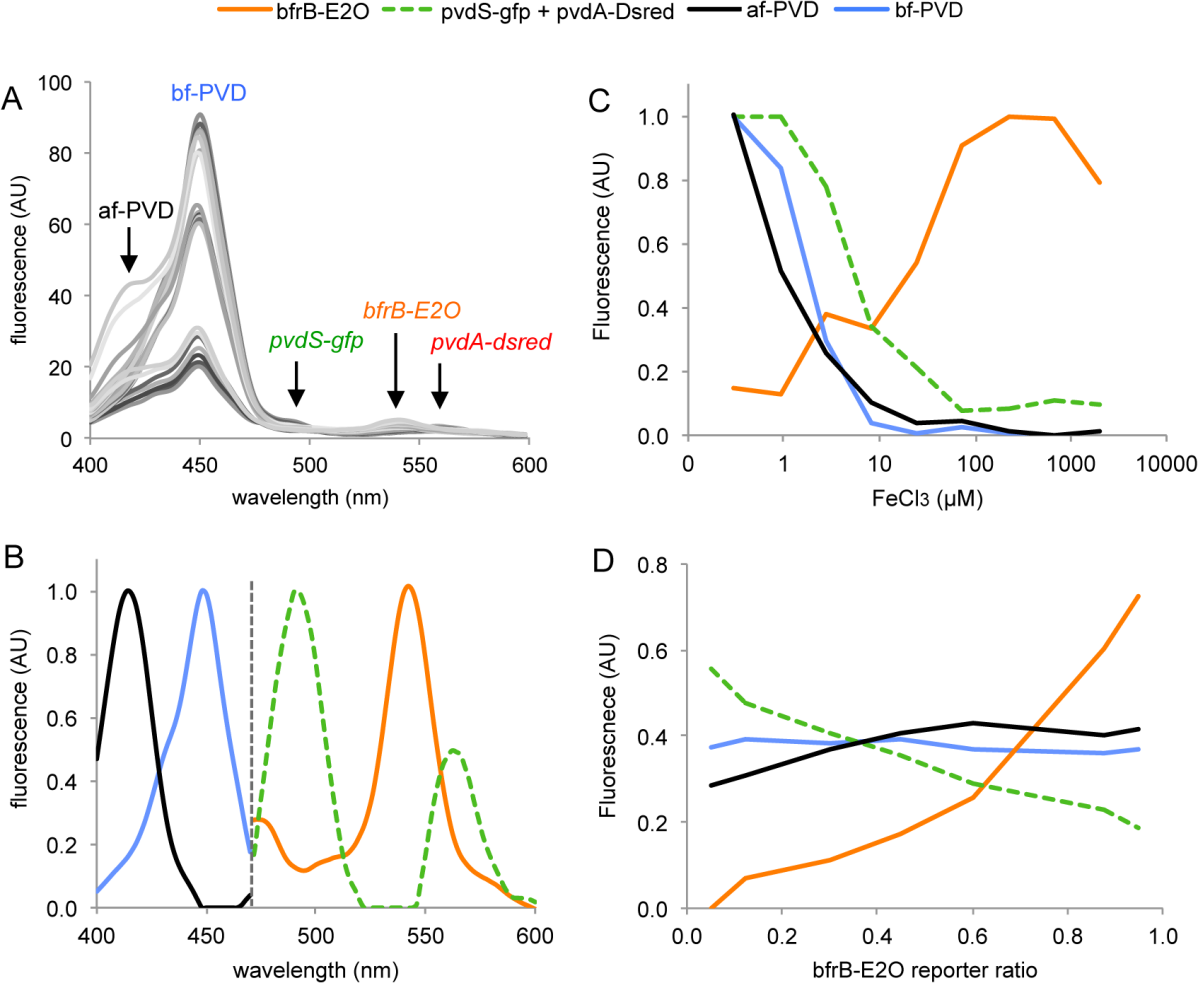
Spectral decomposition of fluorescence from mixtures of three *P*. *aeruginosa* PAO1 iron bioreporter strains harboring *pvdS-gfp*, *pvdA*-*dsred-express2* and *bfrB-e2-orange* fusions. (A) Raw synchronous spectra of strain mixtures incubated with 0.3, 1 and 3 μM FeCl_3_ showing the relative intensity of fluorescent signals. (B) Spectra of the four fluorescent sources identified from CP analysis performed independently on 400–470 nm and 470–600 nm wavelength ranges. (C) Profile of fluorescence sources as a function of iron concentration. (D) Profile of fluorescence sources as a function of the ratio of *bfrB-e2-orange* reporter strain in the mixture. The mixture pattern for this experiment is presented in S3 Fig. PVD: pyoverdine; af-PVD and bf-PVD: “acid” and “basic” forms of pyoverdine.

Blind CP decomposition of multispectral data is constrained by the level of linear dependencies of mixed fluorescent sources with respect to the tested variables. In case of reduced collinearity, i.e. when the different sources evolve almost independently, as for *pvdA* and *bfrB* that show opposite iron-dependent transcription response, the solution provided by CP decomposition is unique. In case of higher level of collinearity, similar response profiles may lead to incomplete separation by the CP algorithm. In order to test this situation, we repeated the above experiment with an additional *pvdS-gfp* fluorescent construct. The *pvdS* gene encodes an alternative sigma factor that is required for the production of pyoverdine [33]. Like *pvdA*, the expression of *pvdS is regulated* by the ferric uptake regulator (Fur) and is optimal under iron limiting conditions. For the sake of demonstration, cells harboring the *pvdS-gfp* and *pvdA*-*dsred-express2* constructs were intentionally kept in similar proportions to increase collinearity when mixed with the *bfrB-E2-orange* reporter. Mixtures of the three iron bioreporter strains incubated with different iron concentrations were analyzed by synchronous spectroscopy. A rapid examination of raw spectra shows that they are dominated by the pyoverdine signal at low iron concentration (Fig 4A). The signal intensity from *pvdS-gfp* (shoulder peak at 490 nm) and *pvdA-dsred-express2* (peak at 558 nm) did not exceed ca. 10% that of bf-PVD. Owing to this, CP analysis of the whole data set could not separate properly the three collinear sources, which were combined in a single composite spectrum after decomposition (not shown). We therefore performed CP decomposition on spectral data from 470 nm to 600 nm in order to exclude most of the pyoverdine fluorescence from the analysis and avoid biases related to their intense signals. This procedure enabled the separation of the GFP signal from bf-PVD, but not from DsRed-express2 (Fig 4B). As expected, both the green and red signals were estimated as a single source due to high degree of collinearity. Nevertheless, they displayed a typical iron response profile (Fig 4C), close to that obtained with *pvdA* in the previous experiment. The E2-Orange signal due to *bfrB* expression was identified as well, and showed the expected iron-dependent induction profile, with however a less marked decrease occurring around 10 μM FeCl_3_. Finally, CP decomposition of spectral data from 400 to 470 nm enabled the identification of the two forms of pyoverdine.

Results from this experiment indicate that proper CP decomposition of multiple fluorescence sources can be affected both by their level of collinearity and their intensity. Incomplete separation of sources from fluorescent reporters, as observed with *pvdS* and *pvdA*, is not a problem per se. Rather it indicates that genes follow the same expression pattern. In contrast, low intensity signals may not be discriminated from disproportionally high ones, or even unidentified when too close to the background noise level. Indeed, attempts to include a fourth PAO1 strain expressing mCherry constitutively in the above mixtures led to a further dilution of the fluorescent signals produced by the reporters and the lowest one (DsRed-Express2) could not be properly identified (not shown).

## Conclusions

This work introduces a spectroscopic approach for multiplex fluorescent bacterial biosensing. We show that synchronous fluorescence spectroscopy offers a simple and efficient means not only of narrowing and simplifying spectra of FPs, but also of recording signals from FP mixtures in a single scan. Application of the popular Candecomp-Parafac decomposition tool to synchronous spectral array data obtained from artificial mixtures of FP labeled *E*. *coli* strains led to successful blind identification of each FP component. The method proved to be sufficiently sensitive for the analysis of three *P*. *aeruginosa* iron bioreporters mixtures with low fluorescence output. Moreover, it revealed subtle yet unexpected iron-dependent bacterioferritin gene expression and enabled concomitant detection of pyoverdine and a related fluorescent signals that would not have been detected otherwise.

Our method is implemented in standard microplates, a convenient 2D format to examine the simultaneous influence of two variables on the expression of several fluorescent reporters and to generate three-way (variable 1 x variable 2 x wavelength) spectral data that can be analyzed with CP. It should be noted that time can be used as a variable in this system, thereby enabling the temporal dynamics of multiple reporter gene expression to be monitored as well. Applications that can be envisioned include, for instance, characterizing the behavior of natural and synthetic genetic networks [34], [35], monitoring the activity of different fluorescent labeled bacteria in complex environmental samples [36] or the detection of multiple pollutants in a same sample [37]. Furthermore, the approach is not restricted to the analysis of genetically encoded fluorescent signals and could be expanded, as demonstrated here with the detection of pyoverdine, to any compound that emits a quantifiable fluorescence such as organic dyes or quantum dots. Although the method provides a qualitative determination of mixed fluorescents signals at the moment, the expansion to quantitative analysis is achievable and currently under development using the standard addition method or through incorporation of an internal fluorescent standard.

## Supporting Information

S1 Fig Microplate dispensing pattern of FP labeled *E*. *coli* strains. Seven serially diluted cell suspensions of *E*. *coli* TOP10 producing GFP, TurboYFP (YFP), E2-Orange (E2O) and DsRed-Express2 (DsRx) were mixed in five different ratios to simulate variation of FP expression. The untransformed TOP10 strain was used as a diluent to maintain a constant cell concentration (i.e. 2.10^8^ cells/well) in the wells. The number of each fluorescent bacteria per well is given. Color-graded triangles represent the dilution level of each fluorescent strain from column 1 to 7 as well as from line A to E.
(TIF)

S2 Fig Microplate dispensing pattern of two iron responsive bioreporter strains. Cell suspensions of PAO1/pPB-bfrB-O561 and PAO1/pPB-pvdA-R591 (approx. 4.10^7^ cells/ ml) were mixed in seven different ratios (95:5; 85:15; 75:25; 50:50; 25:75; 15:85 and 5:95 from lane A to G) and supplemented with indicated concentrations of FeCl_3_ from column 1 to 12.
(TIF)

S3 Fig Microplate dispensing pattern of three iron responsive bioreporter strains. Cell suspensions of PAO1/pPROBE-NT’-pvdS, PAO1/pPB-bfrB-O561 and PAO1/pPB-pvdA-R591 (approx. 4.10^7^ cells/ml) were mixed in seven different ratios (47.5:47.5:5; 47.5:40:12.5; 40:30:30; 30:25:45; 20:20:60; 5:10:85 and 2.5:2.5:95 from lane A to G) and supplemented with indicated concentrations of FeCl_3_ from column 1 to 9.
(TIF)

S1 Text Methods for vector construction. (DOCX)

## References

[1] Chudakov DM, Matz MV, Lukyanov S, Lukyanov KA. Fluorescent Proteins and Their Applications in Imaging Living Cells and Tissues. Physiol Rev. 2010;90: 1103–1163. 10.1152/physrev.00038.2009 20664080

[2] Larrainzar E, O’Gara F, Morrissey JP. Applications of autofluorescent proteins for in situ studies in microbial ecology. Annu Rev Microbiol. 2005;59: 257–277. 10.1146/annurev.micro.59.030804.121350 16153170

[3] Ghim C-M, Lee SK, Takayama S, Mitchell RJ. The art of reporter proteins in science: past, present and future applications. BMB Rep. 2010;43: 451–460. 20663405 10.5483/bmbrep.2010.43.7.451

[4] Van der Meer JR, Belkin S. Where microbiology meets microengineering: design and applications of reporter bacteria. Nat Rev Microbiol. 2010;8: 511–522. 10.1038/nrmicro2392 20514043

[5] Shaner NC, Steinbach PA, Tsien RY. A guide to choosing fluorescent proteins. Nat Methods. 2005;2: 905–909. 10.1038/NMETH819 16299475

[6] Day RN, Davidson MW. The fluorescent protein palette: tools for cellular imaging. Chem Soc Rev. 2009;38: 2887–2921. 10.1039/b901966a 19771335 PMC2910338

[7] Zimmermann T. Spectral Imaging and Linear Unmixing in Light Microscopy In: Rietdorf J, editor. Microscopy Techniques. Springer Berlin Heidelberg; 2005 pp. 245–265. Available: 10.1007/b102216 16080271

[8] Bansal L, Nelson R, Yang E, Jayaraman A, Hahn J. Experimental design of systems involving multiple fluorescent protein reporters. Chem Eng Sci. 2013;101: 191–198. 10.1016/j.ces.2013.06.021

[9] Neher RA, Mitkovski M, Kirchhoff F, Neher E, Theis FJ, Zeug A. Blind source separation techniques for the decomposition of multiply labeled fluorescence images. Biophys J. 2009;96: 3791–3800. 10.1016/j.bpj.2008.10.068 19413985 PMC2711419

[10] Harshman RA. Foundations of the PARAFAC procedure: Models and conditions for an “explanatory” multi-modal factor analysis [Internet]. Ann Arbor: UCLA; 1970 pp. 1–84. Report No.: 16. Available: http://publish.uwo.ca/harshman/abstract.html

[11] Bro R. PARAFAC. Tutorial and applications. Chemom Intell Lab Syst. 1997;38: 149–171. 10.1016/S0169-7439(97)00032-4

[12] Sidiropoulos ND, Bro R. On the uniqueness of multilinear decomposition of N-way arrays. J Chemom. 2000;14: 229–239. 10.1002/1099-128X(200005/06)14:3<229::AID-CEM587>3.0.CO;2-N

[13] Miron S, Dossot M, Carteret C, Margueron S, Brie D. Joint processing of the parallel and crossed polarized Raman spectra and uniqueness in blind nonnegative source separation. Chemom Intell Lab Syst. 2011;105: 7–18. 10.1016/j.chemolab.2010.10.005

[14] Guo X, Miron S, Brie D, Stegeman A. Uni-mode and Partial Uniqueness Conditions for CANDECOMP/PARAFAC of Three-Way Arrays with Linearly Dependent Loadings. SIAM J Matrix Anal Appl. 2012;33: 111–129. 10.1137/110825765

[15] Shirakawa H, Miyazaki S. Blind spectral decomposition of single-cell fluorescence by parallel factor analysis. Biophys J. 2004;86: 1739–1752. 10.1016/S0006-3495(04)74242-3 14990501 PMC1304009

[16] Lloyd J. Synchronized excitation of fluorescence emission spectra. Nature. 1971;231: 64–65.

[17] Patra D, Mishra AK. Recent developments in multi-component synchronous fluorescence scan analysis. TrAC Trends Anal Chem. 2002;21: 787–798. 10.1016/S0165-9936(02)01201-3

[18] Visca P, Colotti G, Serino L, Verzili D, Orsi N, Chiancone E. Metal regulation of siderophore synthesis in Pseudomonas aeruginosa and functional effects of siderophore-metal complexes. Appl Environ Microbiol. 1992;58: 2886–2893. 1444402 10.1128/aem.58.9.2886-2893.1992 PMC183023

[19] Ochsner UA, Wilderman PJ, Vasil AI, Vasil ML. GeneChip expression analysis of the iron starvation response in Pseudomonas aeruginosa: identification of novel pyoverdine biosynthesis genes. Mol Microbiol. 2002;45: 1277–1287. 12207696 10.1046/j.1365-2958.2002.03084.x

[20] Miller WG, Leveau JHJ, Lindow SE. Improved gfp and inaZ broad-host-range promoter-probe vectors. Mol Plant Microbe Interact. 2000;13: 1243–1250. 10.1094/MPMI.2000.13.11.1243 11059491

[21] Andersen JB, Sternberg C, Poulsen LK, Bjorn SP, Givskov M, Molin S. New unstable variants of green fluorescent protein for studies of transient gene expression in bacteria. Appl Environ Microbiol. 1998;64: 2240–2246. 9603842 10.1128/aem.64.6.2240-2246.1998 PMC106306

[22] Strack RL, Bhattacharyya D, Glick BS, Keenan RJ. Noncytotoxic orange and red/green derivatives of DsRed-Express2 for whole-cell labeling. Bmc Biotechnol. 2009;9: 32 10.1186/1472-6750-9-32 19344508 PMC2678115

[23] Lutz R, Bujard H. Independent and Tight Regulation of Transcriptional Units in Escherichia Coli Via the LacR/O, the TetR/O and AraC/I1-I2 Regulatory Elements. Nucleic Acids Res. 1997;25: 1203–1210. 10.1093/nar/25.6.1203 9092630 PMC146584

[24] Choi KH, Kumar A, Schweizer HP. A 10-min method for preparation of highly electrocompetent Pseudomonas aeruginosa cells: Application for DNA fragment transfer between chromosomes and plasmid transformation. J Microbiol Methods. 2006;64: 391–397. 10.1016/j.mimet.2005.06.001 15987659

[25] Cormack BP, Valdivia RH, Falkow S. FACS-optimized mutants of the green fluorescent protein (GFP). Gene. 1996;173: 33–38. 10.1016/0378-1119(95)00685-0 8707053

[26] Strack RL, Strongin DE, Bhattacharyya D, Tao W, Berman A, Broxmeyer HE, et al A noncytotoxic DsRed variant for whole-cell labeling. Nat Methods. 2008;5: 955–957. 10.1038/nmeth.1264 18953349 PMC4107390

[27] Shaner NC, Campbell RE, Steinbach PA, Giepmans BNG, Palmer AE, Tsien RY. Improved monomeric red, orange and yellow fluorescent proteins derived from Discosoma sp red fluorescent protein. Nat Biotechnol. 2004;22: 1567–1572. 10.1038/nbt1037 15558047

[28] Andrews SC, Robinson AK, Rodríguez-Quiñones F. Bacterial iron homeostasis. FEMS Microbiol Rev. 2003;27: 215–237. 10.1016/S0168-6445(03)00055-X 12829269

[29] Wilderman PJ, Sowa NA, FitzGerald DJ, FitzGerald PC, Gottesman S, Ochsner UA, et al Identification of tandem duplicate regulatory small RNAs in Pseudomonas aeruginosa involved in iron homeostasis. Proc Natl Acad Sci U S A. 2004;101: 9792–9797. 10.1073/pnas.0403423101 15210934 PMC470753

[30] Tiburzi F, Imperi F, Visca P. Intracellular levels and activity of PvdS, the major iron starvation sigma factor of Pseudomonas aeruginosa. Mol Microbiol. 2008;67: 213–227. 10.1111/j.1365-2958.2007.06051.x 18047579

[31] Romanowski K, Zaborin A, Fernandez H, Poroyko V, Valuckaite V, Gerdes S, et al Prevention of siderophore- mediated gut-derived sepsis due to P. aeruginosa can be achieved without iron provision by maintaining local phosphate abundance: role of pH. BMC Microbiol. 2011;11: 212 10.1186/1471-2180-11-212 21943078 PMC3195088

[32] Valeur B, Berberan-Santos MN. Structural Effects on Fluorescence Emission Molecular Fluorescence. Wiley-VCH Verlag GmbH & Co. KGaA; 2012 pp. 75–107. Available: 10.1002/9783527650002.ch4

[33] Cunliffe HE, Merriman TR, Lamont IL. Cloning and characterization of pvdS, a gene required for pyoverdine synthesis in Pseudomonas aeruginosa: PvdS is probably an alternative sigma factor. J Bacteriol. 1995;177: 2744–2750. 7751284 10.1128/jb.177.10.2744-2750.1995 PMC176945

[34] Cox R, Dunlop M, Elowitz M. A synthetic three-color scaffold for monitoring genetic regulation and noise. J Biol Eng. 2010;4: 10 10.1186/1754-1611-4-10 20646328 PMC2918530

[35] Ball DA, Lux MW, Graef RR, Peterson MW, Valenti JD, Dileo J, et al. Co-design in synthetic biology: a system-level analysis of the development of an environmental sensing device. Pac Symp Biocomput Pac Symp Biocomput. 2010; 385–396. 19908391

[36] Hewitt BM, Singhal N, Elliot RG, Chen AYH, Kuo JYC, Vanholsbeeck F, et al Novel Fiber Optic Detection Method for in Situ Analysis of Fluorescently Labeled Biosensor Organisms. Environ Sci Technol. 2012;46: 5414–5421. 10.1021/es300164p 22502724

[37] Jouanneau S, Durand M-J, Courcoux P, Blusseau T, Thouand G. Improvement of the identification of four heavy metals in environmental samples by using predictive decision tree models coupled with a set of five bioluminescent bacteria. Environ Sci Technol. 2011;45: 2925–2931. 10.1021/es1031757 21355529

