# Correction: Multicolor Whole-Cell Bacterial Sensing Using a Synchronous Fluorescence Spectroscopy-Based Approach

**DOI:** 10.1371/journal.pone.0127211

**Published:** 2015-05-01

**Authors:** The PLOS ONE Staff

The mu character (μ) is incorrectly typeset as a one-quarter character (¼) throughout this article. The publisher apologizes for the errors.

In the “Bacterial strains, plasmids and growth conditions” subsection of the “Materials and Methods” section, the last sentence of the first paragraph should read: When required, the medium was supplemented with kanamycin at final concentrations of 40 μg/ml (for *E*. *coli*) or 250 μg/ml (for *P*. *aeruginosa*).

In the “Mixture of P. aeruginosa iron bioreporter strains” subsection of the “Materials and Methods,” the second sentence should read: The two cell suspensions were then mixed in a black polypropylene 96-well microplate and supplemented with different dilution of FeCl_3_ with an automated pipetting system (Eppendorf epMotion 5070) in a final volume of 200 μl as indicated in S2 Fig.

In the “Application of the joint SFS/CP approach to study iron homeostasis in *P*. *aeruginosa*” subsection of the “Results and Discussion” section, the twelfth sentence should read: However, the iron-dependent induction profile of *bfrB-e2-orange* showed a modest yet reproducible decrease around 25 μM FeCl_3_.

In the “Testing the limits of the SFS/CP approach” subsection of the “Results and Discussion,” the penultimate sentence of the first paragraph should read: The E2-Orange signal due to *bfrB* expression was identified as well, and showed the expected iron-dependent induction profile, with however a less marked decrease occurring around 10 μM FeCl_3_.

The second sentence of the caption for Fig 4 should read: (A) Raw synchronous spectra of strain mixtures incubated with 0.3, 1 and 3 μM FeCl_3_ showing the relative intensity of fluorescent signals. Please see the correct Fig 4 caption below.

**Fig 4:**
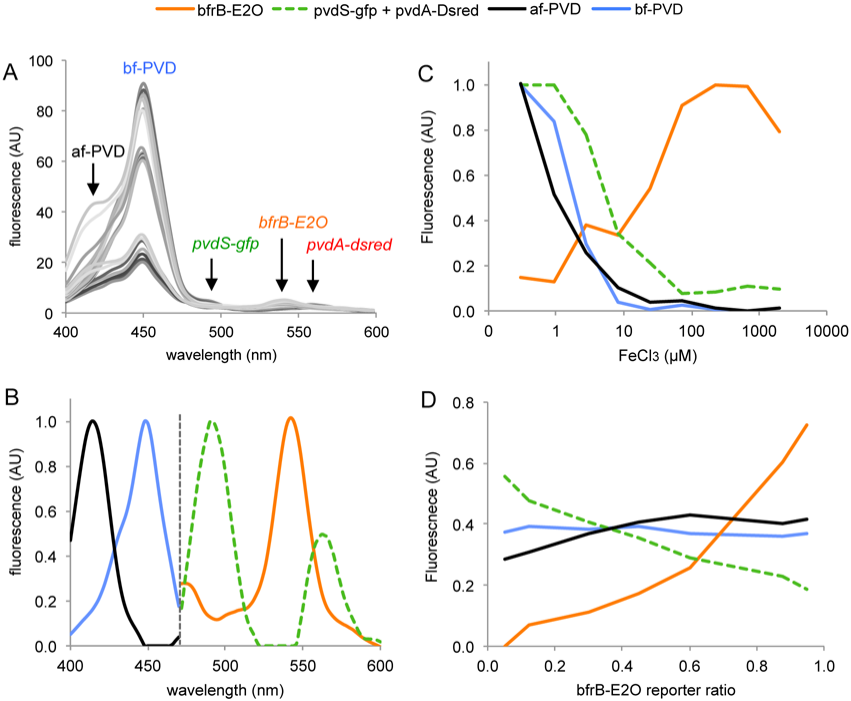
Spectral decomposition of fluorescence from mixtures of three *P*. *aeruginosa* PAO1 iron bioreporter strains harboring *pvdS-gfp*, *pvdA*-*dsred-express2* and *bfrB-e2-orange* fusions. (A) Raw synchronous spectra of strain mixtures incubated with 0.3, 1 and 3 μM FeCl_3_ showing the relative intensity of fluorescent signals. (B) Spectra of the four fluorescent sources identified from CP analysis performed independently on 400–470 nm and 470–600 nm wavelength ranges. (C) Profile of fluorescence sources as a function of iron concentration. (D) Profile of fluorescence sources as a function of the ratio of *bfrB-e2-orange* reporter strain in the mixture. The mixture pattern for this experiment is presented in S3 Fig. PVD: pyoverdine; af-PVD and bf-PVD: “acid” and “basic” forms of pyoverdine.
