# Correction: Multicolor Whole-Cell Bacterial Sensing Using a Synchronous Fluorescence Spectroscopy-Based Approach

**DOI:** 10.1371/journal.pone.0129078

**Published:** 2015-07-01

**Authors:** The PLOS ONE Staff

## Notice of Republication

This article was republished on May 5, 2015, to correct errors throughout the paper that were introduced during the typesetting process. The publisher apologizes for the errors. Please download this article again to view the correct version. The originally published, uncorrected article and the republished, corrected article are provided here for reference. This republication has addressed the issues in the posted correction.

## Supporting Information

S1 File Originally published, uncorrected article. (PDF)

S2 File Republished corrected article. (PDF)
