# Supplementary figures and images for: Multicolor Whole-Cell Bacterial Sensing Using a Synchronous Fluorescence Spectroscopy-Based Approach
Source: PLoS One. 2015 Mar 30;10(3):e0122848. doi: 10.1371/journal.pone.0122848 (PMC4379052; doi:10.1371/journal.pone.0122848)

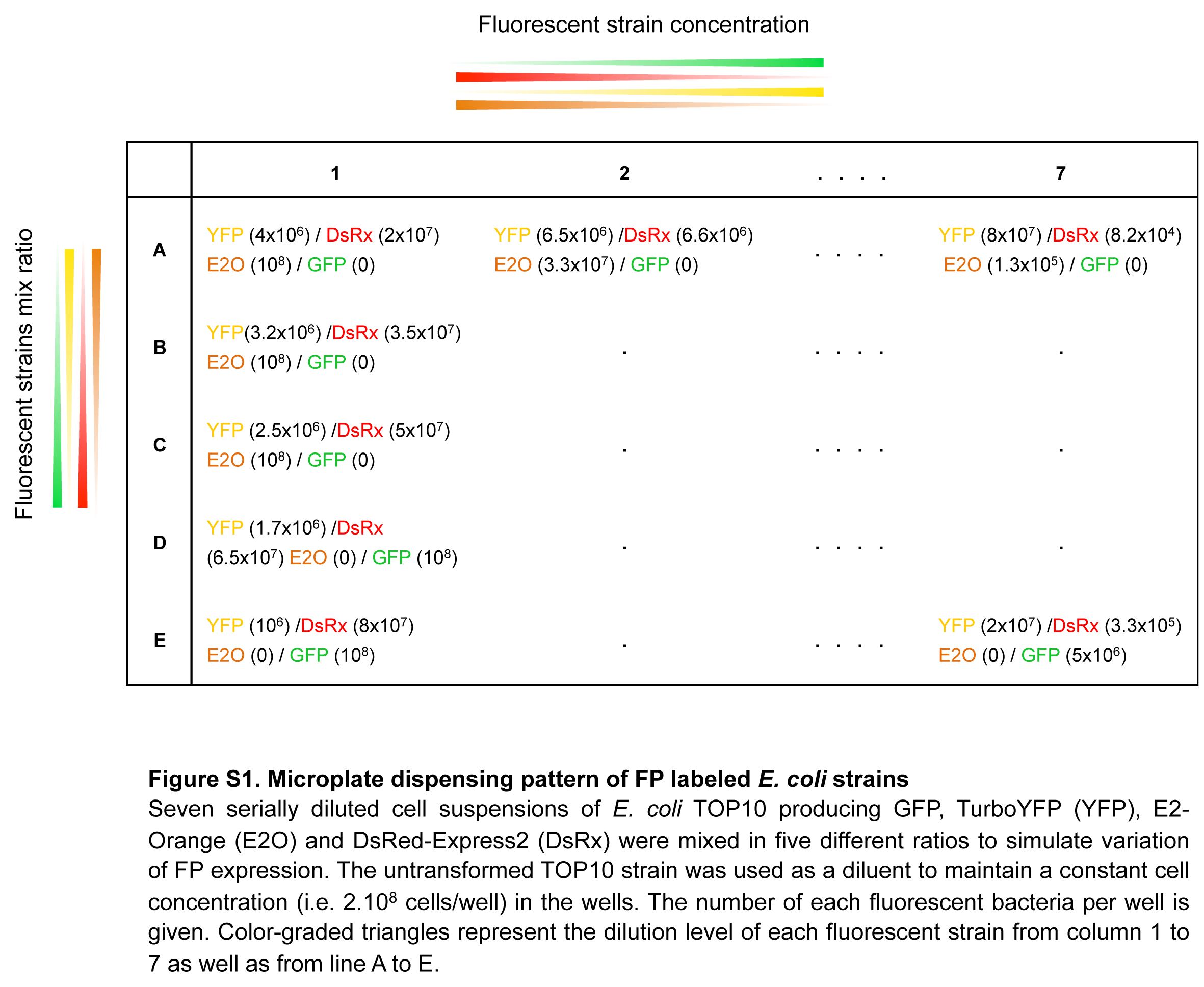

Supplement: S1 Fig — Seven serially diluted cell suspensions of E. coli TOP10 producing GFP, TurboYFP (YFP), E2-Orange (E2O) and DsRed-Express2 (DsRx) were mixed in five different ratios to simulate variation of FP expression. The untransformed TOP10 strain was used as a diluent to maintain a constant cell concentration (i.e. 2.108 cells/well) in the wells. The number of each fluorescent bacteria per well is given. Color-graded triangles represent the dilution level of each fluorescent strain from column 1 to 7 as well as from line A to E. (TIF) [file pone.0122848.s001.tif]

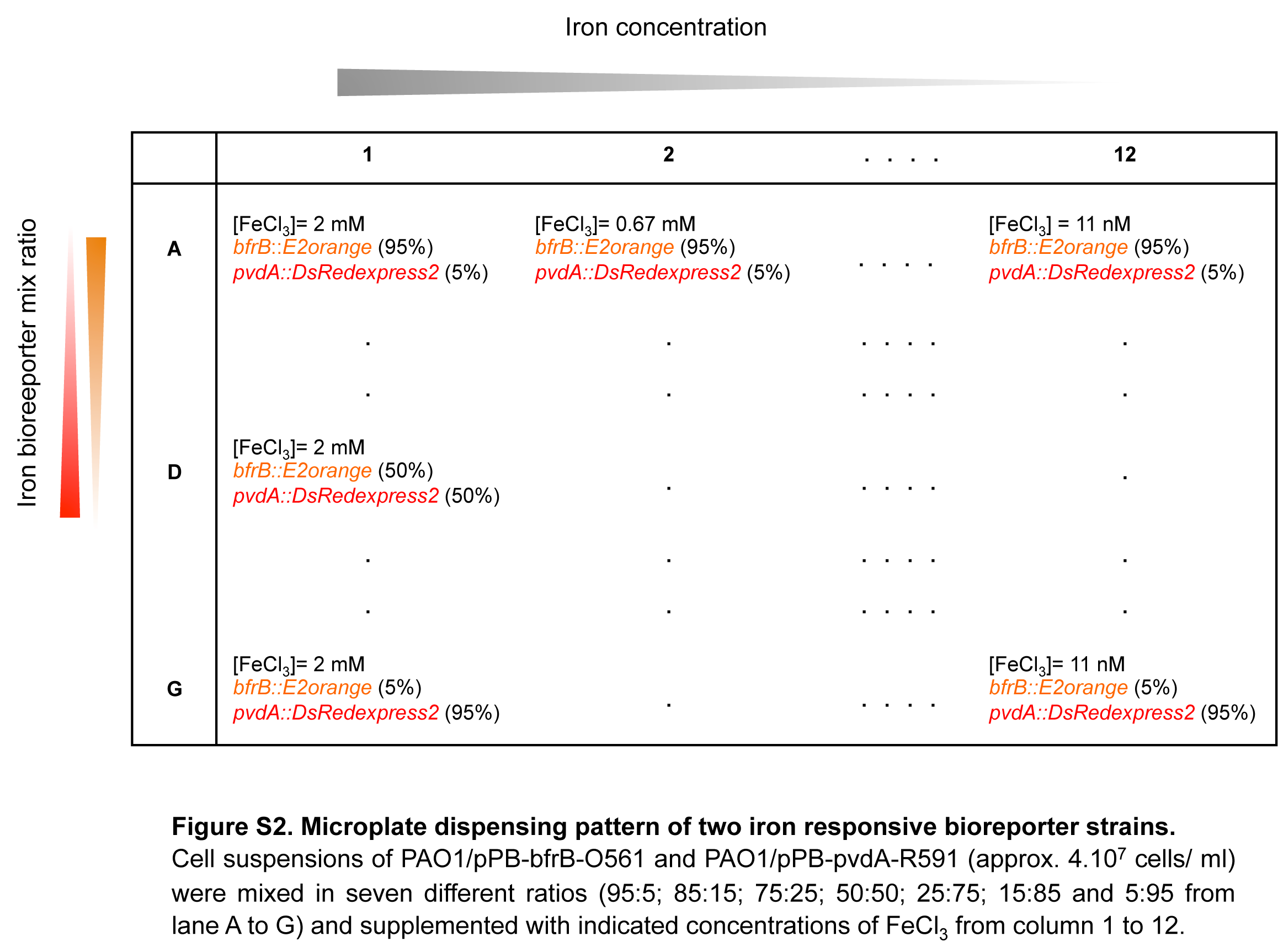

Supplement: S2 Fig — Cell suspensions of PAO1/pPB-bfrB-O561 and PAO1/pPB-pvdA-R591 (approx. 4.107 cells/ ml) were mixed in seven different ratios (95:5; 85:15; 75:25; 50:50; 25:75; 15:85 and 5:95 from lane A to G) and supplemented with indicated concentrations of FeCl3 from column 1 to 12. (TIF) [file pone.0122848.s002.tif]

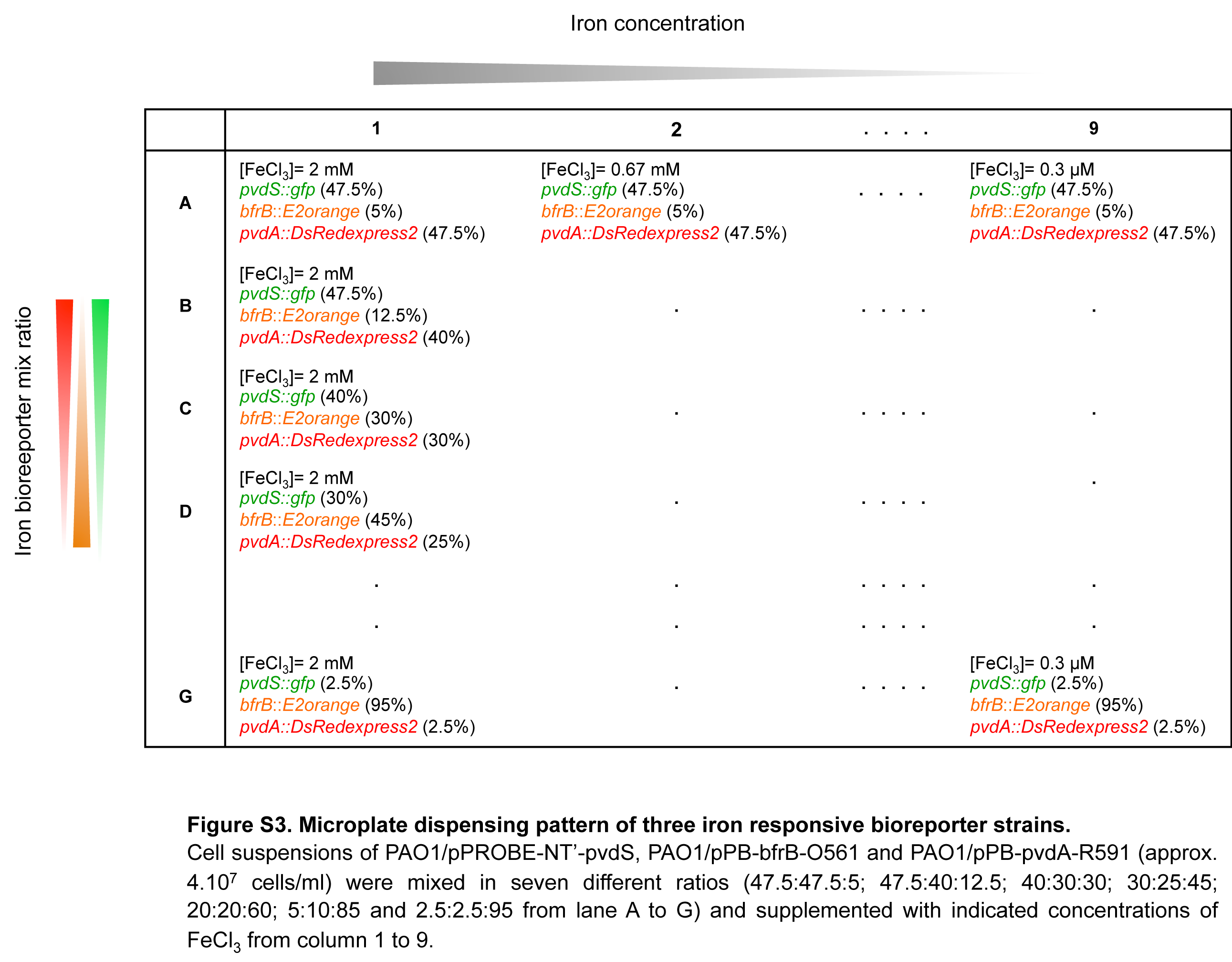

Supplement: S3 Fig — Cell suspensions of PAO1/pPROBE-NT’-pvdS, PAO1/pPB-bfrB-O561 and PAO1/pPB-pvdA-R591 (approx. 4.107 cells/ml) were mixed in seven different ratios (47.5:47.5:5; 47.5:40:12.5; 40:30:30; 30:25:45; 20:20:60; 5:10:85 and 2.5:2.5:95 from lane A to G) and supplemented with indicated concentrations of FeCl3 from column 1 to 9. (TIF) [file pone.0122848.s003.tif]
