# Supplementary material for: Multicolor Whole-Cell Bacterial Sensing Using a Synchronous Fluorescence Spectroscopy-Based Approach
Source: PLoS One. 2015 Mar 30;10(3):e0122848. doi: 10.1371/journal.pone.0122848 (PMC4379052; doi:10.1371/journal.pone.0122848)
Supplement: S1 Text — (DOCX) [file pone.0122848.s004.docx]

**Text S1 : methods for vector construction**

Plasmid pPROBE’-GFP[LVA] [1] served as a backbone for the construction of the pPB plasmid series. For that purpose, we followed a strategy similar to that described by Rochat et al. [2]. The *mcherry* gene was amplified with primer mCheSXFor (5’-GGGCGTCGACTCTAGACTAACTAACTAAAGATTAACTTTATA*AGGAGG*AAAAA CATATGGTGAGCAAGGGCGAGGAG) including SalI, XbaI, NdeI restriction sites (underlined) and a ribosome binding site (italics) and primer mCheHRev (5’-CTCAAGCTTATTACTTGTACAGCTCGTCCATG) including a HindIII restriction site, from plasmids pmcherry (Clontech). The PCR products was digested with SalI and HindIII and inserted in pPROBE’-GFP-[LVA] in place of the *gfp* gene, creating pPBR610. The E2-Orange encoding gene was amplified from pE2-Orange-N1 (Addgene plasmid 21719) with primers pE2NFor (5’-CTCCATATGGATAGCACTGAGAACGTCA) and pE2HRev (CTCAAGCTTACTACTGGAACAGGTGGTG), digested with NdeI and HindIII and cloned into pPBO610 in place of the *mcherry* gene, yielding pPBO561. Genes encoding TurboYFP and DsRed-Express2 were synthesized de novo after codon optimization for *E. coli* (Eurofins MWG operon) and cloned as above NdeI-HindIII fragments into pPBO610, resulting in plasmids pPBY538 and pPBR591, respectively.

To construct plasmids pPBlac-FP, the LacI-repressible *P_A1/O4/O3_* promoter, also referred to as PA1lacO-1 [3] was amplified from pJBA28 using primers P3 (5’-CAGGTACCAT TTATCAGGGTTATTGTCT) and P4 (5’-GGTCTAGATGTGTGAAATTG TTATCCG) and inserted as a KpnI-XbaI fragment upstream the fluorescent protein gene in pPB plasmids. The *P_A1/O4/O3_* PCR fragment was also cloned into pCR2.1 (Invitrogen) and further subcloned into EcoRI-digested pPROBE-NT’ to generate pPROBE-NT’lac.

Iron sensitive reporter plasmids were obtained as follows: the promoter region of the *bfrB* gene from *P. aeruginosa* PAO1 (PA3530-PA3531 intergenic region) was amplified by PCR with bfrB-for (5’-TCGAATTCTCGTCGCTGCCTGAAAAAT) and bfrB-rev (5’- CTCGTCGACTCTTGTTGAGGTGCTGGATG) primers containing EcoRI and SalI restriction sites respectively (underlined) using genomic DNA as a matrix. The 260-bp amplicon was then digested by the SalI and EcoRI and cloned into the pPBO561 plasmid digested with the same enzymes, yielding pPB-bfrB-O561. Similarly, the promoter of *pvdA* (PA2385-PA2386 intergenic region) was amplified by PCR with pvdA-for (5’- CTCGAATTCTGATTTCGCTATTCGTGCTC) and pvdA-rev (5’-CTCGTCGACTTCCA GTTCCTCTGGATTGG) and cloned into SalI and EcoRI-digested pPBR591 to produce pPB-pvdA-R591. The promoter region of *pvdS* was PCR-amplified using primer pair pvdS-For (5’-CCGGAATTCATGACTGCAACATTGGCGC) and pvdS-rev (5’-CTCGTCGACTCTTCACCA GGATCGTACGG) and ligated into EcoRI and SalI-digested pPROBE-NT’ to yiel pPROBE-NT’-pvdS.

**References**

1. Miller WG, Leveau JHJ, Lindow SE (2000) Improved gfp and inaZ broad-host-range promoter-probe vectors. Mol Plant Microbe Interact 13: 1243–1250. doi:10.1094/MPMI.2000.13.11.1243.

2. Rochat L, Pechy-Tarr M, Baehler E, Maurhofer M, Keel C (2010) Combination of fluorescent reporters for simultaneous monitoring of root colonization and antifungal gene expression by a biocontrol pseudomonad on cereals with flow cytometry. Mol Plant-Microbe Interact MPMI 23: 949–961. doi:10.1094/MPMI-23-7-0949.

3. Lutz R, Bujard H (1997) Independent and Tight Regulation of Transcriptional Units in Escherichia Coli Via the LacR/O, the TetR/O and AraC/I1-I2 Regulatory Elements. Nucleic Acids Res 25: 1203–1210. doi:10.1093/nar/25.6.1203.
